# Suspected HSV vs. demyelinating transverse myelitis in patient on TNF alpha inhibitor: a case report

**DOI:** 10.3389/fmed.2026.1787999

**Published:** 2026-05-08

**Authors:** Sarah Mahfouz, Tania Loutfi, Wafaa Jreige, Hanna Mattar

**Affiliations:** School of Medicine and Medical Sciences, Holy Spirit University of Kaslik, Byblos, Lebanon

**Keywords:** CNS infection, immunosuppressive therapy, neuroimmune disorders, neurological complications, transverse myelitis

## Abstract

Herpes reactivation is a rare complication in patients receiving immunosuppressive medications, particularly tumor necrosis factor (TNF) inhibitors, particularly when it manifests as myelitis. Meanwhile, a first episode of transverse myelitis is closely related to the early risk of developing a demyelinating disease such as multiple sclerosis (MS) and MS-like conditions. We report the case of a middle eastern man in his early 30s who was previously diagnosed with spondyloarthritis, currently treated with adalimumab, and who developed cervical myelitis, showing as a T2 hypersignal on magnetic resonance imaging (MRI) localized at the level of his C2 vertebra with a unilateral clinical presentation, concomitant with positive serology testing for herpes simplex virus (HSV), suggestive of a reactivation, in the absence of a feasible cerebrospinal fluid (CSF) analysis. Management was primarily through methylprednisolone pulse therapy and intravenous (IV) acyclovir, in addition to physical rehabilitation and the temporary discontinuation of adalimumab, yielding positive results and a full recovery at 6 months. This case highlights the importance of early detection and treatment of neuroimmune complications in patients receiving immunosuppressive therapy, using non-invasive and minimally invasive techniques. It also showcases the complexity of differentials for acute transverse myelitis when no sufficient evidence is available for a neuroinfectious or demyelinating disease diagnosis.

## 1 Introduction

Tumor necrosis factor (TNF) inhibitors, such as adalimumab, are largely used in the treatment of rheumatic diseases and other chronic inflammatory conditions such as ankylosing spondylitis and spondyloarthritis (1). Unfortunately, a downside to their efficacy is potent suppression of the host’s immune defenses, which would predispose to an increased risk of reactivation of latent pathogens, such as a latent herpes simplex virus (HSV) infection or a varicella zoster virus (VZV) infection (2, 3). Transverse myelitis is an uncommon neuroinflammatory disorder, which can manifest as a motor and sensory deficit, with or without autonomic dysregulation. Even though symptoms are typically bilateral, unilateral presentations may occur (2, 4). Myelitis itself is a rare manifestation of herpetic reactivation, with only very few cases documented in the literature. Aside from infectious causes, transverse myelitis should also prompt investigations for demyelinating diseases, particularly in the context of a broader autoimmune predisposition (4–6).

### 1.1 Clinical presentation and history

A Middle Eastern male patient in his early 30s presented to our emergency department for unilateral paresthesia and weakness in his left upper extremity, progressively worsening over the past week. The paresthesia, which initially began distally at the tip of his fingers in a pattern more evocative of a median nerve compression, began extending proximally, including the entirety of his left upper limb, further extending his left retro-auricular space (C2-C3 territory) and descending ipsilaterally to his neck and trunk, reaching the level of T10 at the umbilicus by the day of his presentation. No other neurological findings were noted at the time. There was no fever, no rash, and no associated cardio-pulmonary, urinary, or gastrointestinal symptoms and no symptoms suggestive of an autonomic dysfunction.

The patient has a history of spondyloarthritis, diagnosed approximately 8 years ago, and initially treated with methotrexate (15 mg twice a week), and subsequently transitioned to adalimumab (40 mg subcutaneously every 2 weeks) as his current therapy, on which he was generally stable with no recent flare-ups. His spondyloarthritis initially manifested as bilateral elbow and knee enthesitis, with no radiological findings on the spine prior to his current presentation. His past medical history is also notable for hypertension, dyslipidemia, pericarditis (11 years ago), hepatic steatosis, and bilateral Achilles tendinitis. Based on his past surgical history, he successfully underwent surgery for Morton’s neuroma 10 years prior, a tonsillectomy, and a nasal polypectomy. He has no history of smoking, has history of occasionally drinking alcohol, and is allergic to latex. The patient reported no positive family history of autoimmune diseases or other relevant chronic conditions and has no recent travel history.

### 1.2 Physical examination

In the emergency department, his vitals were stable, with a blood pressure of 140/70 mmHg, a heart rate of 77 bpm, a temperature of 36.4 °C, and O_2_ saturation of 98% in room air. On physical examination, the patient was awake, alert, and oriented. His motor power was estimated at 5/5 on the right, 2/5 in his left upper extremity, and 3/5 in his left lower extremity. Light touch sensation was fully preserved on the right but decreased on the left side of his upper body, with worse distal sensory function. All deep tendon reflexes were normal, evaluated at 2 + bilaterally. No alteration in proprioception or cerebellar functions. Cranial nerve exam was normal, and the rest of his examination was unremarkable.

### 1.3 Workup

**Figure 1:**
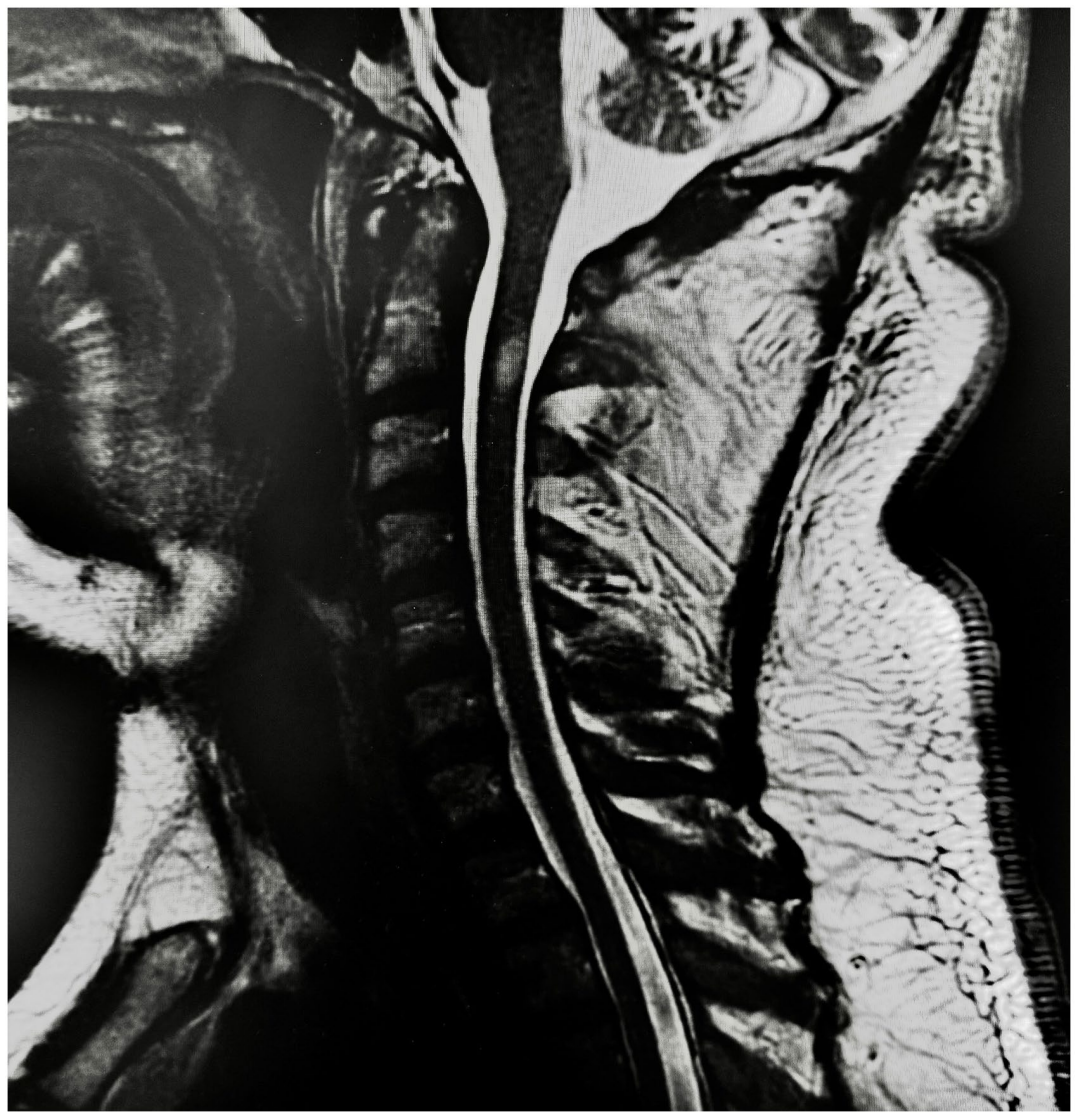
MRI of the cervical spine, T2-weighted sagittal sequence, showing a 6 mm segment of hyperintensity involving the posterolateral spinal cord at the level of C2, suggests transverse myelitis.

Cerebral and cervical magnetic resonance imaging (MRI) was performed, revealing left posterolateral transverse myelitis at the level of C2, extending more than 6 mm, with diffuse bone demineralization, as depicted in Figure 1. The cerebral MRI revealed no pertinent findings. The patient was admitted for further etiological investigations and adequate treatment.

As some sources recommend performing a full spine MRI in the case of a transverse myelitis due to the potential non-correlation of clinical symptoms to the location of the inflammatory process (7), a thoracic and lumbar MRI were performed upon admission, showing an L5-S1 disco-ligamental posterolateral protrusion that is non-compressive but of significant thickness, as well as multiple non-specific bone modifications of the lumbar spine, which may suggest ankylosing spondylitis. As a result, two attempts at a lumbar puncture failed, despite ultrasound guidance. Fluoroscopy guidance was not available to us at the time. With these limitations precluding cerebrospinal fluid (CSF) analysis, the focus shifted to serological testing.

The most substantial serological findings were positive IgG and IgM HSV antibodies, which, together with the radiological data, may suggest herpetic reactivation at the level of the cervical spine. Other serological testing uncovered positive Epstein–Barr virus (EBV) and cytomegalovirus (CMV) IgG with negative IgM antibodies. Antibodies to VZV and human herpesvirus 6, 7, and 8 were negative. The purified protein derivative (PPD) test for tuberculosis was negative. Aside from a white blood count of 17,180, the complete blood count was normal, and vitamin B12 levels were normal but near the lower limit. The rest of his workup was unremarkable, including electrolytes, urinalysis, lactate dehydrogenase (LDH), thyroid-stimulating hormone (TSH), and a low C-reactive protein (CRP) level of 3. Hepatic and pancreatic workup values were unchanged compared to the patient’s baseline.

### 1.4 Management and evolution

Management measures first included the discontinuation of immunosuppressive medications. The patient was started on a course of IV methylprednisolone pulse therapy for 4 days and intravenous (IV) acyclovir at a dose of 15 mg/Kg every 8 h for 14 days. Daily physical rehabilitation was initiated alongside symptomatic treatment.

Within the first 48 h of hospitalization, before the initiation of acyclovir treatment, the patient’s sensory and motor deficit progressed to also involve his left lower extremity (L4 territory). A few days after the initiation of IV acyclovir and methylprednisolone pulse therapy, he developed a vesicular rash visible on his left upper limb, neck, and chest. Polymerase chain reaction (PCR) testing was highly considered, ideally to be performed within the first 24 h after the rash appeared, but this was unfortunately not possible due to the unavailability of PCR testing at our institution, as well as administrative and financial reasons, which precluded taking the sample for analysis elsewhere. The rash later resolved after a full course of acyclovir. Within 2 weeks of IV treatment and physiotherapy, the patient regained more than 60% of his lost motor function and sensory function. He was later discharged on pregabalin 75 mg 1 tablet (tab) twice daily for a month, followed by 1 tablet every night for a month; vitamin B12 5,000 1 tablet sublingually once a week for 2 months, in addition to his routine medications, and he was encouraged to continue physical therapy. A few weeks later, he resumed his daily activities with a satisfactory recovery. Assessment at 6 months showed full recovery with a return to his functional baseline. Follow-up visits with his neurologists were scheduled on a regular basis to monitor his myelitis for the risk of demyelinating disease progression.

**Figure 2:**
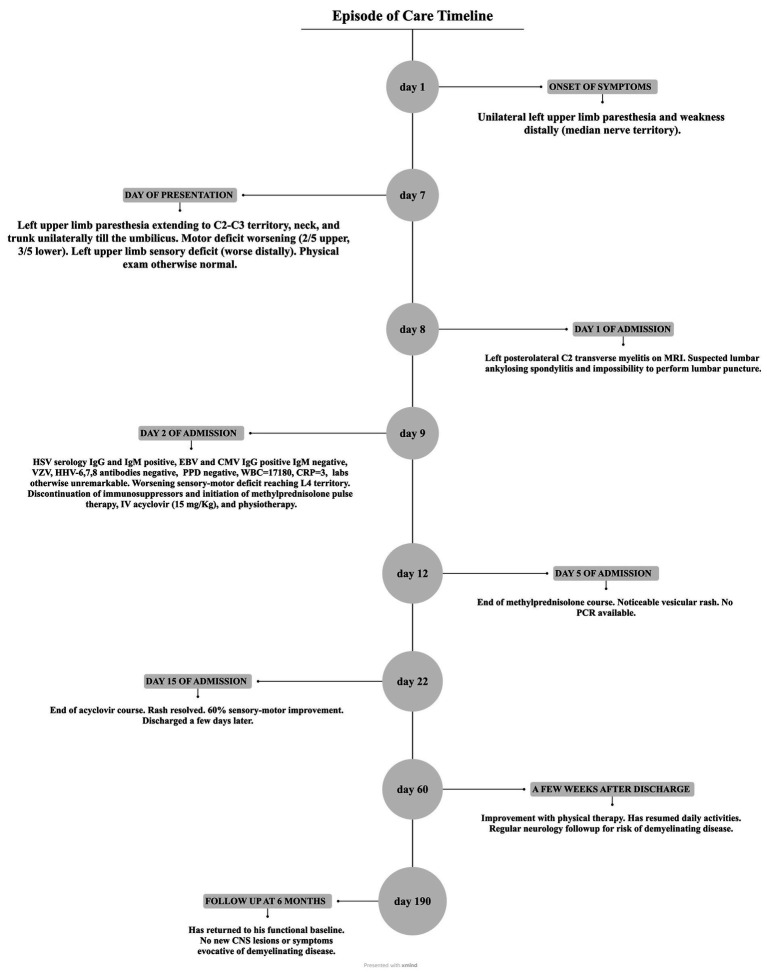
Case timeline during episode of care from onset of symptoms to follow-up at 6 months.

Notably, the patient’s clinical presentation and evolution are summarized in Figure 2, along with key events that defined the timeline of his care.

## 2 Discussion

Posterolateral myelitis, which was discovered on cervical MRI, given its neuroanatomical location and the patient’s clinical progression, could explain the occurring symptoms. However, establishing an etiology for the inflammation remains essential. With the gathered findings, the patient’s history, and given the diagnostic limitations of this case, differential diagnoses were narrowed down to the following working theories: The first, being the reactivation of an underlying herpes simplex infection, manifests as a rare occurrence of herpes simplex myelitis, with only a few cases being reported throughout the literature (7–9). The second, an etiology that correlates with the long-standing immunosuppressive therapy, since TNF inhibitors, such as adalimumab, were described as being associated in rare cases with the occurrence of myelitis (10, 11), with the assumption that an altered immune system would predispose to an environment that is more vulnerable to infectious pathogens, particularly of herpes viruses (2). While the third hypothesis involves the patient’s own autoimmune predisposition, given that spondyloarthritis could precipitate the development of myelitis (12) and could predispose the patient to having other autoimmune conditions, namely multiple sclerosis (MS) and MS-like diseases.

To tackle the demyelinating aspect of the differential diagnoses for this case, it is important to note that, given this first presentation of a symptomatic transverse myelitis—uncovered as a single lesion on MRI and symptoms lasting more than 24 h—this case could qualify at least as a clinically isolated syndrome (CIS) according to the 2017 McDonald criteria (13). If HSV serological testing alone is considered to be insufficiently accurate, particularly given the unavailability of CSF and PCR testing (14) and the lack of infectious symptoms initially at presentation, this does not preclude the classification of the case from being considered a CIS. However, because CSF oligoclonal bands and kappa free light chains were not obtainable, there is a lack of sufficient evidence to consider this case anything beyond CIS according to the 2024 McDonald criteria for MS at the time of events (15). This, of course, does not eliminate the risk of uncovering an underlying MS or MS-like condition with future disease progression, hence prompting close follow-up, particularly given the high risk of MS associated with both transverse myelitis itself (4) and with the use of TNF alpha inhibitors (16). An additional point is that EBV testing was particularly relevant to this differential, beyond its role in the neuroinfectious workup for myelitis, given the reported evidence linking EBV to MS; therefore, EBV IgG positivity in this case also indicates a higher risk for MS (17). As for other demyelinating diseases on the differential diagnoses, we note neuromyelitis optical spectrum disorder (NMOSD) (4, 5) and myelin oligodendrocyte glycoprotein antibody-associated disease (MOGAD) (5, 6), which would typically display a more aggressive initial presentation, a more extensive myelitis, and/or optic nerve involvement (6), as opposed to what is observed in our case. AQP4 and MOG antibodies will be tested in the future as part of a broader MS and MS-like workup, in case of further disease suspicion on follow-up.

Given the diagnostic limitations at the time of events, an outcome-based approach to initial acute management was adopted, consisting of the methylprednisolone and acyclovir trial therapy. In fact, the first-line treatment for transverse myelitis is IV glucocorticoids (4). This combination accounts for both a potential demyelinating disease, which is typically addressed in the acute phase with IV steroids, and an HSV contribution, as there are currently no absolute guidelines for the treatment of HSV myelitis specifically; however, the tendency is to treat the underlying infection with 10–15 mg/kg of acyclovir for 10–14 days, up to 21 days in cases of associated encephalitis, often in combination with methylprednisolone pulse therapy for 2–4 days or 60–80 mg of oral prednisone for 3–5 days (4, 5, 7, 8). Alternative options include plasma exchange or IV immunoglobulins, which are used for transverse myelitis refractory to IV steroids (4, 5). On a separate note, vitamin B12 supplementation was added to the treatment upon discharge as an adjunct to provide symptomatic relief of neuropathic symptoms, given that B12 levels were at the lower limit. Those levels were monitored at follow-up visits after completion of the short-term supplementation course and were within the normal range, which prompted no further supplementation or investigations for the moment.

To mention that a broader infectious workup for other pathogens with reported associations to transverse myelitis, including enteroviruses, West Nile virus, HIV, Zika virus, human T-cell leukemia virus type 1 (HTLV-1), *Treponema pallidum* (neurosyphilis), and *Borrelia burgdorferi* (Lyme neuroborreliosis) (4), were not performed acutely since their index of suspicion was low as per patient history and exposure, and the main treatment was going to rely essentially on IV methylprednisolone, as previously mentioned. Testing for these pathogens was nonetheless noted in case of further indications at any point in the course of the disease.

## 3 Patient perspective

I have been affected by an inflammation of the spinal cord, which has caused me partial paralysis. This neurological condition has impacted several areas of my body, particularly my neck, left upper limb, and left lower limb, up to my knee. I have gone through periods of intense physical pain and nerve pain. Since the beginning of this ordeal, I have been living with reduced mobility, requiring regular care, rehabilitation, and constant adaptation to this new physical reality. Despite the daily challenges this represented, I maintained confidence and courage, relying on medical, spiritual, and family support to return to my daily life.

## 4 Conclusion

Finally, in the context of autoimmune diseases and multiple comorbidities and risk factors, uncommon patterns have been observed and should be taken in account, particularly when the index of suspicion is high, despite possible limitations of certain diagnostic tools. This includes neuroimmune complications such as transverse myelitis, prompting early detection and adequate management of potential infectious and autoimmune etiologies. Such an approach could substantially impact the patient’s recovery and quality of life.

## Data Availability

The datasets presented in this article are not readily available because of ethical and privacy restrictions. Requests to access the datasets should be directed to the corresponding author.
